# Transcriptional Profiling of Egg Allergy and Relationship to Disease Phenotype

**DOI:** 10.1371/journal.pone.0163831

**Published:** 2016-10-27

**Authors:** Roman Kosoy, Charuta Agashe, Alexander Grishin, Donald Y. Leung, Robert A. Wood, Scott H. Sicherer, Stacie M. Jones, A. Wesley Burks, Wendy F. Davidson, Robert W. Lindblad, Peter Dawson, Miriam Merad, Brian A. Kidd, Joel T. Dudley, Hugh A. Sampson, M. Cecilia Berin

**Affiliations:** 1 Department of Genetics and Genomic Sciences, Icahn School of Medicine at Mount Sinai, New York, NY, United States of America; 2 Department of Pediatrics, Icahn School of Medicine at Mount Sinai, New York, NY, United States of America; 3 Department of Pediatrics, National Jewish Health, Denver, CO, United States of America; 4 Department of Pediatrics, Johns Hopkins University School of Medicine, Baltimore, MD, United States of America; 5 Department of Pediatrics, University of Arkansas for Medical Sciences and Arkansas Children's Hospital, Little Rock, AR, United States of America; 6 Department of Pediatrics, University of North Carolina, Chapel Hill, NC, United States of America; 7 National Institute of Allergy and Infectious Diseases, NIH, Bethesda, MD, United States of America; 8 EMMES Corporation, Rockville, MD, United States of America; 9 Oncological Sciences, Icahn School of Medicine at Mount Sinai, New York, NY, United States of America; Harvard Medical School, UNITED STATES

## Abstract

**Background:**

Egg allergy is one of the most common food allergies of childhood. There is a lack of information on the immunologic basis of egg allergy beyond the role of IgE.

**Objective:**

To use transcriptional profiling as a novel approach to uncover immunologic processes associated with different phenotypes of egg allergy.

**Methods:**

Peripheral blood mononuclear cells (PBMCs) were obtained from egg-allergic children who were defined as reactive (BER) or tolerant (BET) to baked egg, and from food allergic controls (AC) who were egg non-allergic. PBMCs were stimulated with egg white protein. Gene transcription was measured by microarray after 24 h, and cytokine secretion by multiplex assay after 5 days.

**Results:**

The transcriptional response of PBMCs to egg protein differed between BER and BET versus AC subjects. Compared to the AC group, the BER group displayed increased expression of genes associated with allergic inflammation as well as corresponding increased secretion of IL-5, IL-9 and TNF-α. A similar pattern was observed for the BET group. Further similarities in gene expression patterns between BER and BET groups, as well as some important differences, were revealed using a novel Immune Annotation resource developed for this project. This approach identified several novel processes not previously associated with egg allergy, including positive associations with TLR4-stimulated myeloid cells and activated NK cells, and negative associations with an induced Treg signature. Further pathway analysis of differentially expressed genes comparing BER to BET subjects showed significant enrichment of IFN-α and IFN-γ response genes, as well as genes associated with virally-infected DCs.

**Conclusions:**

Transcriptional profiling identified several novel pathways and processes that differed when comparing the response to egg allergen in BET, BER, and AC groups. We conclude that this approach is a useful hypothesis-generating mechanism to identify novel immune processes associated with allergy and tolerance to forms of egg.

## Introduction

Egg allergy is an IgE-mediated food allergy that affects 0.5–2.5% of young children, although a population-based study from Australia showed that 8.9% of 12-month-old infants are reactive to raw egg [1]. Egg allergy is highly associated with eczema and with peanut allergy. There are different clinical phenotypes of egg allergy. The majority of children who are allergic to egg can tolerate extensively heated forms of egg (baked egg), and their prognosis is better than those who react to extensively heated forms of egg [2]. A similar phenotypic difference has been described for milk allergy [3, 4]. There is some literature to suggest that these two forms of egg allergy are immunologically distinct. Children who are reactive to extensively heated egg have greater levels of ovalbumin- and ovomucoid-specific IgE, and larger skin-prick-test wheals to egg than children who are tolerant of extensively heated egg [5]. Analysis of the epitopes recognized by IgE indicate that children with persistent egg allergy uniquely recognize linear epitopes that are maintained in heat denatured proteins [6]. Children with tolerance to heated forms of milk have been shown to have an increased frequency of milk-specific Tregs [7], suggesting that there may be active regulatory components to this form of clinical tolerance.

Specific hypotheses about the role of egg-responsive CD4+ T cells in allergy and immunotherapy-induced tolerance are being tested in ongoing clinical trials. We proposed that an unbiased transcriptional profiling approach would be informative in identifying novel immune pathways contributing to the pathogenesis of egg allergy, and potentially in discriminating phenotypes of egg allergy. Transcriptional profiling of PBMCs has been used in juvenile idiopathic arthritis and systemic lupus erythematosus to identify disease-related processes, importantly leading the way to new therapeutic approaches (i.e. the use of the IL-1 receptor antagonist anakinra in juvenile idiopathic arthritis) [8, 9]. Molecular heterogeneity in lupus has also been uncovered using such approaches [10] which paves the way for tailored treatment approaches. We examined the transcriptional response to egg antigen of PBMCs from children with two phenotypes of egg allergy (tolerant vs. responsive to baked egg) and food allergic controls that were clinically tolerant to egg. Our approach was successful in identifying novel immune pathways associated with egg allergy. In addition to identifying associations with individual genes and their products, such as TNFα and IL-9, we identified several novel processes including TLR4-activated myeloid cell gene signatures that were associated with the response to egg in PBMCs from egg allergic subjects. Furthermore, we observed that regulation of IFN-α and -γ response genes differed between individuals with reactivity or tolerance to baked egg. Future studies will determine the contribution of these pathways to the BER and BET phenotypes.

## Methods

### Participants

Baseline blood samples were obtained from children aged 3–16 years at time of enrollment (prior to egg exposure) in a multi-center intervention trial comparing the efficacy of baked egg or egg oral immunotherapy (OIT) in the treatment of egg allergy (CoFAR7, NCT01846208). These participants included children with an egg-specific IgE > 5 kU/L who had confirmed reactions to baked egg (baked egg responsive, BER), or were tolerant to baked egg but had confirmed reactions to (unheated) egg white protein as determined by a double-blind placebo-controlled food challenge (baked egg tolerant, BET). BER and BET subjects were avoiding egg at the time of enrollment. Atopic pediatric controls (AC) were recruited from the Jaffe Food Allergy Institute at the Icahn School of Medicine at Mount Sinai (ages 5–19). These subjects were food allergic (to peanut (n = 7), tree nuts (n = 7) and fish (n = 1)), had low levels of detectable IgE to egg, and were clinically tolerant to egg. Peanut-specific IgE was measured in this group, ranging from <0.1 kU/L (in a cashew-allergic subject) to >100 kU/L (in 4 of 7 peanut-allergic subjects). Of 10 AC subjects tested for levels of peanut-specific IgE, 9 were sensitized to peanut with a median of 21.8 kU/L (range 0.48 - >100 kU/L). **Table 1** summarizes the characteristics of these subjects, including egg-specific IgE levels. The National Institute of Allergy and Infectious Diseases Allergy and Asthma Data and Safety Monitoring Board, and the Institutional Review Boards at Icahn School of Medicine at Mount Sinai, National Jewish Health, Johns Hopkins Medical School, University of Arkansas for Medical Sciences, and University of North Carolina, Chapel Hill approved study procedures. Written informed consent was obtained from next of kin, caretakers, or guardians on behalf of minors/children enrolled in the study.

**Table 1:** Study group demographics and sample number.

		Group	AC	BET	BER
		N	14	21	38
		Age*	11.4 (5–19)	7.8 (5–16)	8.2 (6–15)
		Egg-IgE (kU/L)*	1.56 (<0.01–11.4)	26.9 (5.13–239)	64.8 (4.3–497)
Sample Availability	Supernatants (After QC)	Media	12	14	23
		EW	12	15	28
		Paired	12	14	23
	RNA (After QC)	Media	14	19	31
		EW	14	20	34
		Paired	14	19	31

*Values are mean(range)

### Blood Samples

Blood samples were obtained in 10 ml sodium-heparin Vacutainer tubes at the 5 clinical sites. Whole blood was shipped overnight in temperature-controlled Greenbox^TM^ shipping containers (ThermoSafe, Arlington Heights, IL) assembled according to standard operating procedures. Temperature loggers were included to ensure that temperatures were maintained between 20 and 30°C. Samples from the Icahn School of Medicine at Mount Sinai clinical site were stored at room temperature and processed the next day to maintain consistency with the other sites.

### Cell Isolation and Stimulation

PBMCs were isolated from whole blood by Ficoll-Paque PLUS (GE Healthcare, Uppsala, Sweden), washed, and cultured in AimV (ThermoFisher, Grand Island,NY) with 2.5% autologous plasma. 4 x 10^6^ cells were plated in 1 ml in 24-well culture plates, in the presence or absence of 300 μg of egg white (EW) protein (Deb-El Food Products, Elizabeth, NJ). EW had been cleaned of endotoxin using Detoxi-Gel columns (ThermoFisher). Residual endotoxin levels were approximately 0.3 EU/ml of working solution of egg stimulant (99.85% removal, and within the acceptable range for culture reagents). The dose of antigen was based on pilot experiments conducted to optimize detection of egg-responsive T cells by flow cytometry. Cells were cultured in standard tissue culture incubators for 24 h prior to harvest of RNA, or 5 days prior to harvest of culture supernatants for cytokine level detection.

### Cytokine Measurement

Secreted cytokines were measured using ProcartaPlex Human Cytokine Panel 1B (25-plex) (eBioscience, San Diego, CA) using Luminex microsphere technology. The supernatants were processed according to manufacturer’s instructions, and read on a Luminex® 200™ System with data processing via xPONENT 3.0 version (Luminex, Austin, Tex). The relationship between EW stimulation dependent cytokine levels and clinical groups was evaluated using the difference between cytokine levels after EW vs. M stimulations (EW-M). The significance of the difference between the log10 transformed EW-M cytokine levels (in pg/ml) and clinical groups was evaluated with univariate robust linear regression using rlm function from MASS package in R, with the three clinical levels defined as a categorical variable with AC as a baseline. FDR correction was applied to the nominal p-values using Benjamini–Hochberg procedure using p.adjust function in stats package in R.

### RNA Isolation and Microarray Analysis

Medium was removed and cells harvested for RNA isolation using the Qiagen RNeasy mini kit (Valencia, CA) according to manufacturer’s instructions. RNA was kept at -80°C until used for microarray analysis. Gene expression was measured in five separate batches using Illumina Human HT-12v3 and HT-12v4 Expression BeadChip Array (Illumina, San Diego, CA) following vendor recommended protocol, starting with 200 nanogram of good quality total RNA. For biotinylated cRNA generation Ambion Illumina TotalPrep RNA Amplification Kit was used following manufacturer’s recommended protocol, and the cRNA was hybridized to the Human HT-12 v4 bead array. The cRNA hybridized to the array was labeled with Cy3 dye and scanned on Illumina HiScan array scanner, and the microarray signals were processed with Genome Studio Gene Expression Module GSGX Version 1.9.0 (Illumina, San Diego, IL, USA). The raw microarray data were processed by Lumi package in R, with background adjustment, variance-stabilizing transformation, and quantile normalization within each batch. Only the 47231 probes in common between Human HT-12v3 and v4 were retained, of which 2168 were filtered out as fewer than 20 percent of samples had a signal above the background (derived from 770 mismatch probes). The remaining probes were mapped to 19956 human genes, with the probe with the highest expression representing each gene. A total of 11 samples were removed following a QC analysis with a combination of PCA, hierarchical clustering, and XIST/UTY gene based gender check. The remaining samples included samples from 34 BER patients, 20 BET patients, and 14 AC patients.

Since the DEG signatures from different comparisons vary greatly in size, we standardized the size of DEG signatures across conditions, by keeping the top 200 most differentially expressed genes (by fold change) among the top 500 most significant genes).

### Co-Expression Network and Module Analyses

Weighted correlation network analysis and module identification was completed using WGCNA R package [11]. The batch corrected expression for 19959 genes in the 105 samples with available cytokine measurements were utilized for the analysis, with the final network constructed with soft power of 7, minimal module size of 50 genes and the final module cutoff of 0.29, leading to identification of 39 modules. Each module may be represented by a summary profile, or an eigengene, which is effectively a weighted average expression profile of all the genes in the module. Modules consist of genes whose expression is correlated irrespective of the direction of correlation, so some genes in a module may have an opposite pattern of expression from the eigengene representing the module.

The correlation between the modules and the cytokine levels and clinical information was evaluated through linear regression, and correction for multiple testing utilized Benjamini-Hochberg FDR algorithm.

### Functional Enrichment Analyses

Enrichment of co-expression modules and differentially expressed gene signatures was done utilizing Fisher’s exact test on datasets downloaded from public sources: GO, KEGG, REACTOME, and TFT were downloaded from MSigDB (http://software.broadinstitute.org/gsea/msigdb/collections.jsp), and additional transcription factor target sets based on Chip-seq and ChIP-chip experiments were obtained from ChEA dataset (http://amp.pharm.mssm.edu/lib/chea.jsp). Correction for multiple testing utilized Benjamini-Hochberg FDR algorithm.

### Immune Annotation Resource

Starting with the 1910 signatures available in the MSigDB C7 Immunologic signatures set (as of October 2015), individual signatures were grouped into categories based on the shared functional similarity between the two conditions (cell type, stimulation state) used to generate each signature taking the direction of comparison into consideration. The signatures were grouped at a number of different levels, differing on the scale and functional focus. The first Immune Annotation level organized 1763 signatures into 17 categories corresponding to major immune cell populations, for example “CD8_cells” category consisting of 177 signatures. This level can be further subdivided into categories according to the source organism, comprising 1763 signatures in 27 categories, for example “CD8_cells_Human” (15 signatures) and “CD8_cells_Mouse” (162 signatures). The second Immune Annotation level focused on finer subdivision of cell population phenotypes, including the maturation level, tissue location, defined functional sub-populations, comprising 1755 signatures in 131 categories, for example “Induced_Treg” (26 signatures) and “Naive_Spleen_Bcell” (6 signatures). The third Immune Annotation level focuses on the activation state of the immune cell populations, and includes 1862 signatures in 161 categories, with examples being “Acute_Infection_CD8” (2 signatures), “TLR_Stim_Mphage” (42 signatures). In addition, since there was a particular interest to focus on finer delineation of TLR stimulation of myeloid cells, it was possible to further subset categories according to the mechanism of myeloid cell stimulation (5 TLR agonist and anti-TRIM1 antibodies), combining 294 signatures into 22 categories (between 3 and 36 signatures per category) from three GEO datasets (GSE14769, GSE17721, and GSE9988) into Immune Annotation level 4. These included expression signatures with stimulation through TLR4, TLR3, TLR1/TLR2, TLR9, TLR7, and TREM1 antibody in monocytes and macrophages.

The utilization of the Immune Annotation resource is based on enrichment of enrichment approach, where the first stage is enrichment analysis for all relevant individual immunologic signatures (since some Immunological signatures could not be assigned to a relevant category, the numbers of signatures utilized at each level were 1763, 1755, 1862, and 294), and keeping only the signatures which satisfy a particular significance threshold (FDR adjusted pVal ≤ 0.01 used throughout the analysis). In the second stage, each category containing at least one significantly enriched signature was tested to determine whether the category itself is enriched using all significantly enriched signatures as a background. The combined Immune Annotation Score incorporates the enrichment significance per category and the median significance value of enrichments for the significantly enriched signatures within the category.

signatures_median(FDRadjustedpValue)=medianBHadjustedpValuefromFisher’sexacttestforallsignificantlyenrichedsignaturespercategory

category(pValue)=pValueforFisher’sexacttest(onesided)forthecategory’senrichmentforthesignificantlyenrichedsignatureswithinthecategoryusingallsignificantlyenrichedsignaturesasabackground

$$Immune\;Ontology\;Score=\sqrt{-\log_{10}signatures\_median(FDR\;adjusted\;pValue)\;*\;-\log_{10}category(pValue)\;}$$

Since FDR adjusted p-value ≤0.01 was utilized as the threshold for significance of enrichment per individual Immunologic signature, Immune Annotation Score over 2 is observed when the category is significantly enriched for enriched signatures (i.e signatures’ median (FDR adjusted p-value) is ≥2 by default and category’s Fisher’ exact test’s p-value ≤0.01), or when the individual signatures’ median enrichment significance is very high and the category significance is trending toward significance. Alternatively, the category’s FDR adjusted p-value from the Fisher’s exact test can be used on its own as a measure of category’s enrichment.

## Results

### Egg-Induced Cytokine Production Differs According to Egg Allergy Status

Little is known about the immunological response profiles of egg-allergic children who either tolerate (BET) or react to (BER) baked egg. To determine whether these two groups are distinguishable in terms of their response to in vitro stimulation with egg antigen, we measured a spectrum of cytokines secreted by PBMCs following culture for 5 days with EW or medium alone. Similarly treated PBMCs from food allergic but egg tolerant controls (AC) were included for comparison. Comparable patterns of cytokine secretion were observed in the two egg-allergic groups (**Table 2**), with highest levels of cytokines observed in the BER group. Comparisons between the BER and AC groups revealed that out of 18 cytokines that were consistently above the level of detection, eight (GM-CSF, IL-5, IL-7, IL-9, IL-21, IL-31, IFNα, and TNFα) were significantly upregulated (p<0.05) in BER subjects vs. AC subjects, with TNFα and IL-9 remaining significant after FDR correction (BH adjusted p-value ≤ 0.05). TNFα, IL-9, and IL-5 were nominally significantly higher in baked egg tolerant (BET) versus AC subjects. There were no significant differences in cytokine production when comparing the two egg allergic groups (BER vs BET).

**Table 2:** Cytokine secretion by group and stimulation condition.

	AC		BET			BER		
Cytokine	Media	EW	Media	EW	*p (vs AC)*	Media	EW	*p (vs AC)*
GM-CSF	2	11.6	2.9	44.6	*0*.*13*	**4.7**	**83.2**	***0*.*04***
IFNα	0	0	0.9	1.1	*0*.*89*	**0.6**	**1**	***0*.*04***
IFNγ	3.8	203	3	202	*0*.*71*	5.4	591	*0*.*68*
IL-10	12.9	114.2	27.2	132	*0*.*13*	31.7	186	*0*.*41*
IL-13	1	202.5	2.4	1737.5	*0*.*14*	2.4	2186	*0*.*05*
IL-17A	0.4	264	1.1	304	*0*.*69*	1.3	395	*0*.*95*
IL-1a	0.1	0.9	0.5	7.9	*0*.*47*	0.1	3.8	*0*.*16*
IL-1b	1.1	17.5	6.1	65.9	*0*.*10*	5.6	57	*0*.*40*
IL-21	1	1	0.5	14.9	*0*.*06*	**0.5**	**34.7**	***0*.*01***
IL-27	14.7	61.7	7.5	43.5	*0*.*17*	13.6	76.3	*0*.*36*
IL-31	1	1	1	1.3	*0*.*18*	**1**	**2.1**	***0*.*01***
IL-4	1	1	2	5	*0*.*06*	2	6.1	*0*.*07*
IL-5	0.5	54.6	0.5	**252.5**	***0*.*04***	**0.5**	**576**	***0*.*004***
IL-6	0.8	5531	16.6	3201	*0*.*24*	6.7	6904	*0*.*97*
IL-7	0.4	1	0.7	1.3	*0*.*35*	**0.3**	**1**	***0*.*04***
IL-9	3	3	1	**123**	***0*.*003***	**1**	**131**	*0*.001*
TNFα	1	6.8	2	**70.5**	***0*.*03***	**1**	**155**	*0*.0006*
TNF-beta	0.5	49.4	1	49.4	*0*.*82*	1	80.8	*0*.*37*

Bolded values highlight nominal significance

* indicates FDR corrected p value < 0.05.

### Egg-Induced Differential Gene Expression and Association with Clinical Phenotype

We used microarray analysis to measure mRNA expression of 19959 genes in PBMCs after 24h of culture with EW or media in BER, BET and AC subjects. The differentially expressed gene (**DEG**) signatures were identified using three sets of comparisons. In the first set we compared BER to AC, BET to AC, and BER to BET in either EW or media conditions. In the second set we compared the difference between EW and media conditions within groups. The third set compared the differences between stimulation conditions between different clinical groups, i.e. difference of differences (BER_(EWvs.M)_vs.AC_(EWvs.M)_). The last comparison identified genes modulated by exposure to egg to a different extent across different clinical phenotypes. The sizes of DEG signatures generated for each comparison are shown in **Table 3**. EW stimulation induced significant changes in a large number of genes in each clinical group, with the greatest number of genes affected in the BER group, followed by the BET and the AC groups.

**Table 3:** Sizes of up-regulated and down-regulated differentially expressed gene signatures.

Test Set Up		Upregulated Genes^1^		Downregulated Genes	
Stimulation	Group	p-value^2^ < 0.05	FDR p-value (BH)^3^ < 0.05	p-value < 0.05	FDR p-value (BH) < 0.05
EW	BER vs AC	1248	138	1274	104
	BET vs AC	1038	26	1096	24
	BER vs BET	709	0	944	0
M	BER vs AC	959	16	1177	18
	BET vs AC	979	2	752	2
	BER vs BET	485	0	952	1
EW vs M	BER	2200	1340	2338	1270
	BET	2008	1039	2553	1146
	AC	1642	488	1637	386
EW vs M	BER vs AC	731	0	733	1
	BET vs AC	1022	3	1105	2
	BER vs BET	568	0	364	0

^1^From the total of 19959 tested genes

^2^p-value from a multivariate linear model from limma R package

^3^FDR rate from Benjamini–Hochberg adjustment of the p-values done in limma R package

**Fig 1:**
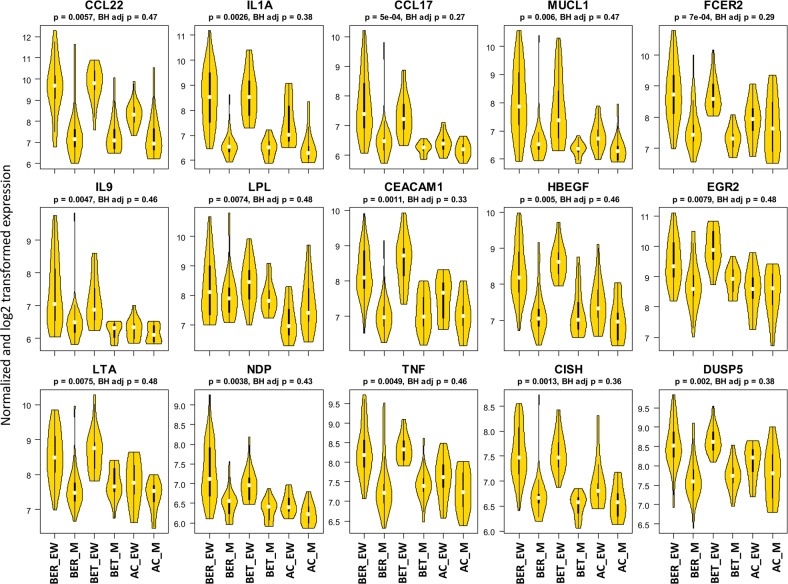
Egg-Induced differential gene expression. Violin plots displaying egg-induced gene expression for the 15 genes that were most differentially expressed when comparing BER to AC subjects. Statistical analysis is indicated after each gene name.

We first focused on transcriptional differences between the most clinically distinct groups (BER vs AC). **Table 4** shows the top 30 up-regulated and down-regulated genes by log2 fold change for the BER_(EWvs.M)_ vs. AC_(EWvs.M)_ comparison, and the top 15 up-regulated genes are shown by group and stimulation condition in **Fig 1**. The products of the up-regulated genes with at least nominal significance (p≤ 0.05) and FDR adjusted p-value ≤ 0.5 (more likely to be true positive than false positive) included several cytokines that we also observed by protein secretion (IL-9, IL-5, and TNFα), as well as Th2-associated chemokines (CCL22, CCL17), IgE receptor (FcεRII), and growth factors and their receptors. Downregulated genes predominantly encode lysosomal and endosomal enzymes and scavenger receptors. The observed upregulation of IL-9, IL-5, CEAMCAM1, and CISH was consistent with another published report of gene expression from purified peanut-responsive CD4+ T cells from peanut allergic individuals [12]. Thus we were able to detect a gene signature consistent with allergen-specific CD4+ T cells using bulk PBMC microarray.

**Table 4:** Top 30 up regulated and down-regulated genes in BER_(EWvs.M)_ vs. AC_(Ewvs.M)_ differential expression sorted by fold change.

	UP-regulated genes	log_2_FC	Ave. expr^1^	p-value^2^	FDR p-value^3^	Down-regulated genes	log_2_FC	Ave. expr	p-value	FDR p-value
1	IL1B^4^	1.22	9.07	0.0099	0.51	FUCA1	-0.97	10.04	0.0145	0.54
2	**CCL22**	**1.07**	**8.41**	**0.0057**	**0.47**	LGMN	-0.89	9.42	0.0155	0.55
3	**IL1A**	**0.99**	**7.45**	**0.0026**	**0.38**	APOE	-0.84	8.59	0.0247	0.6
4	**CCL17**	**0.97**	**6.84**	**0.0005**	**0.27**	**IL18BP**	**-0.82**	**9.3**	**0.0016**	**0.37**
5	**MUCL1**	**0.97**	**7.09**	**0.006**	**0.47**	**CLC**	**-0.79**	**9.81**	**0.002**	**0.38**
6	**FCER2**	**0.95**	**8.08**	**0.0007**	**0.29**	TGFBI	-0.75	10.88	0.0195	0.57
7	PI3	0.83	9.07	0.1089	0.76	GPNMB	-0.74	10.67	0.0504	0.68
8	**IL9**	**0.8**	**6.73**	**0.0047**	**0.46**	CD36	-0.72	9.38	0.0143	0.54
9	TGM2	0.79	8.14	0.0202	0.58	**GJB2**	**-0.67**	**7.08**	**0.0026**	**0.38**
10	IL1RN	0.78	9.27	0.0308	0.63	**DFNA5**	**-0.65**	**8.52**	**0.0003**	**0.27**
11	CCL3	0.77	9.42	0.0435	0.66	THBS1	-0.63	9.39	0.1983	0.84
12	**LPL**	**0.76**	**7.99**	**0.0074**	**0.48**	CCL2	-0.63	11.84	0.0901	0.75
13	CCL3L1	0.73	9.09	0.0474	0.67	CD163	-0.62	9.28	0.0426	0.66
14	**CEACAM1**	**0.67**	**7.64**	**0.0011**	**0.33**	RNASE1	-0.62	9.07	0.2424	0.86
15	**HBEGF**	**0.66**	**7.72**	**0.005**	**0.46**	**OLFML2B**	**-0.62**	**7.39**	**0.0017**	**0.37**
16	CCL4L2	0.65	9.31	0.044	0.66	APOC1	-0.61	8.68	0.0717	0.72
17	CCL3L3	0.65	10.07	0.0613	0.71	**DNASE2**	**-0.61**	**8.66**	**0.0002**	**0.24**
18	CCL20	0.63	7.8	0.0977	0.75	**CTSB**	**-0.61**	**11.21**	**0.0008**	**0.3**
19	**EGR2**	**0.62**	**9.08**	**0.0079**	**0.48**	SPP1	-0.6	9.34	0.2061	0.84
20	ALDH1A2	0.6	8.1	0.0459	0.66	**ANKRD33**	**-0.59**	**9.28**	**0.0091**	**0.5**
21	TNFAIP6	0.59	8.65	0.1291	0.79	SLC16A10	-0.59	7.78	0.0174	0.56
22	QPCT	0.59	8.91	0.0166	0.55	**HAMP**	**-0.58**	**7.1**	**0.001**	**0.32**
23	CCL4L1	0.58	9.48	0.0324	0.64	ADAMDEC1	-0.57	9.38	0.0787	0.73
24	**LTA**	**0.55**	**8.01**	**0.0075**	**0.48**	**GPR162**	**-0.56**	**8.03**	**0.0025**	**0.38**
25	SERPINB2	0.54	9.33	0.2235	0.85	PLD3	-0.54	9.82	0.0146	0.54
26	**NDP**	**0.53**	**6.7**	**0.0038**	**0.43**	MERTK	-0.53	7.83	0.0224	0.59
27	**TNF**	**0.52**	**7.77**	**0.0049**	**0.46**	CSF3R	-0.52	8.34	0.0556	0.69
28	**CISH**	**0.52**	**7.03**	**0.0013**	**0.36**	**TMEM51**	**-0.51**	**9.03**	**0.0032**	**0.41**
29	**DUSP5**	**0.52**	**8.11**	**0.002**	**0.38**	NPL	-0.51	9.04	0.014	0.54
30	**IL5**	**0.51**	**6.61**	**0.0043**	**0.46**	MS4A4A	-0.51	7.75	0.0269	0.61

^1^ Average expression (overall) is log2 transformed and normalized value

^2^ p-value from a multivariate linear model with Gender and Batch as covariates from limma R package

^3^ FDR rate from Benjamini–Hochberg adjustment of the p-values done in limma R package

^4^ Bolded genes indicate nominally significant (p < 0.05) with BH adjusted p-val < 0.5 (more likely to be True positive than False positive).

Our findings (both positive and negative) are unlikely to be due to differences in cell populations in the PBMC population. Flow cytometric analysis of cells in whole blood (including CD4+ and CD8+ T cells, B cells, monocyte subsets, NK cells, NK T cells, eosinophils, neutrophils, cDCs, and pDCs) demonstrated no significant difference in cell subsets between BER and BET subjects (data not shown). Furthermore, differentially expressed genes used egg-stimulated conditions compared to an unstimulated media control for each subject, such that any potential differences in cell populations would be normalized.

### Functional Annotation of Egg-Induced DEG Signatures

**Fig 2:**
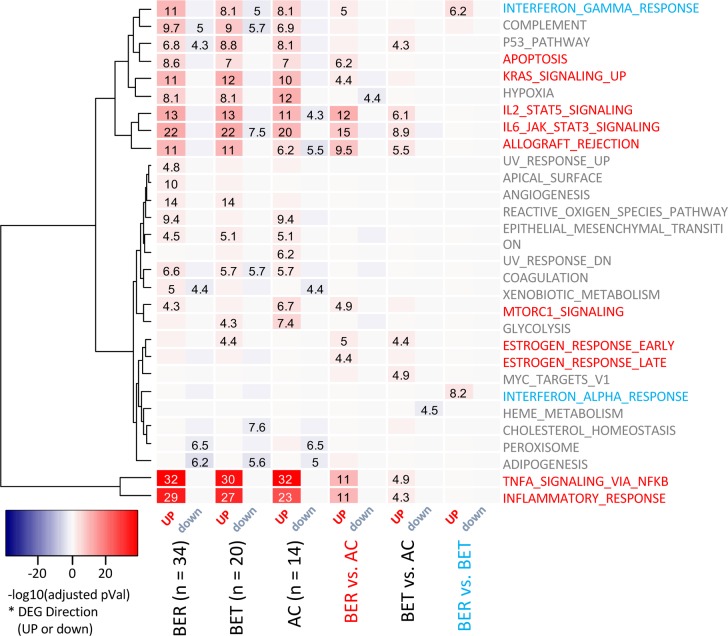
Enrichment of differentially expressed gene signatures in Hallmark functional annotation categories. Enrichment of selected DEG signatures (top 200 by FC from top 500 by p-values) in Hallmark functional categories, with the color intensity indicating–log10 of the BH adjusted p-value and the values are odds ratios from Fisher’s exact tests. The left 3 signatures indicate EW vs. M for each clinical group, while the right 3 signatures indicate the difference in differential (EW vs. M) gene expression between groups. The color indicates whether the genes in the signature were up-regulated (red) or down-regulated (blue). Hallmark categories with at least a single enrichment with BH adjusted p-value ≤ 0.05 (out of total of 50) are shown, clustered according to the similarity of the enrichment pattern. Colored text highlights significant associations with the matching DEG signature.

**Fig 3:**
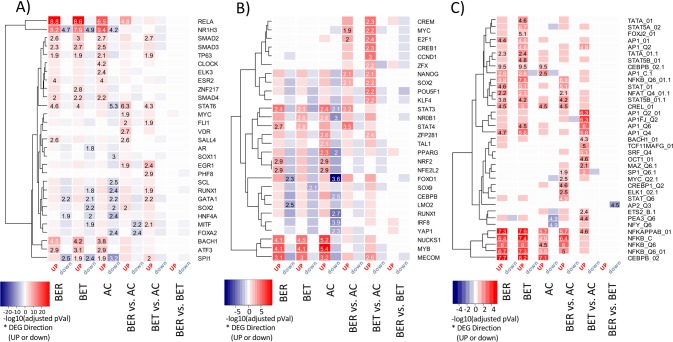
Enrichment of differentially expressed gene signatures for transcription factor targets. Enrichment of the DEG signatures (top 200 by FC from top 500 by p-values) for transcriptional factor target gene sets, with Chip-X (ChIP-chip, ChIP-seq, ChIP-PET and DamID) derived ChEA set from human based experimental data in **A)**, ChEA set from rodent based experimental data in **B)**, and computationally predicted TRANSFACv.1 set in **C)**. The color intensity indicates–log10 of the FDR adjusted p-values and the indicated values are odds ratios (Fisher’s exact test). The color indicates whether the genes in the signature were up-regulated (red) or down-regulated (blue). The results are shown for only twelve (out of the total of 24 used in the analysis) DEG signatures, and only the TF target sets with at least a single enrichment with BH adjusted p-value ≤0.0001 for A) and B) and ≤0.05 for C), clustered according to the similarity of the enrichment pattern.

To assess the functional enrichment with gene signatures, we standardized the size of DEG signatures across the tested comparisons (200 most differentially expressed genes (by fold change) among the top 500 most significant genes). For functional annotation of the DEG signatures, we adopted enrichment approaches utilizing publicly available databases from MSigDB [13]. We focused on curated datasets from Hallmark, GO, KEGG and Reactome, as well as sets of predicted transcriptional factors targets from TRANSFACv1. Additional transcription factor target sets from ChIP-based experiments were obtained from the ChEA dataset for both human and rodent based experimental data [14]. We observed strong enrichment of the signature representing inflammatory response pathways with the DEG signatures up-regulated in both BER and BET groups upon EW stimulation (**Fig 2**). Hallmark signatures representing TNF signaling through NFκB, IL6/JAK/STAT3, and IL2/STAT5 signaling were also significantly enriched in genes up-regulated in BER and BET groups more than in the AC group upon EW exposure. Transcription factors whose targets were most significantly enriched in the up-regulated BER_(EWvs.M)_vs.AC_(EWvs.M)_ DEG signature include RELA (NFKBp65) and STAT6 from the ChEA_human dataset, STAT4 and STAT3 from the ChEA_rodent dataset, and NFKB from the TRANSFAC dataset (**Fig 3**). These are consistent with transcription downstream of cytokines such as IL-4, IL-13, IL-5, IL-9, and TNFα.

**Fig 4:**
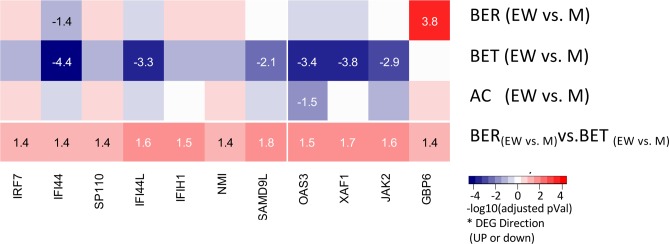
Differential expression of interferon response gene sets. Differential expression of eleven genes from the HALLMARK_ INTERFERON_ALPHA_RESPONSE (including 7 genes also in INTERFERON_GAMMA_RESPONSE, on the left) gene sets overlapping with the DEG signatures (top 200 by FC from top 500 by p-values). Color intensity and values indicate the nominal significance of the differential expression analysis, with values corresponding to negative logarithm of p-values (included for p-values < 0.05). The color indicates whether the genes in the signature were up-regulated (red) or down-regulated (blue).

When we performed enrichment analysis using the BER_(EWvs.M)_ vs.BET_(EWvs.M)_ DEG signature to compare the two egg allergic phenotypes, we observed a unique and significant enrichment of genes in the interferon-γ and interferon-α pathway (**Fig 2**). The BER_(EWvs.M)_vs.BET_(EWvs.M)_ DEG signatures contained 11 genes from the HALLMARK_INTERFERON_GAMMA_RESPONSE gene set (of which 7 genes are also in HALLMARK_INTERFERON_ALPHA_RESPONSE gene set). These genes were selectively down-regulated in the BET group upon EW exposure, while no consistent changes EW-induced changes were observed in the BER or AC patients (**Fig 4**). Expression of interferon receptor IFNGR2 was increased after egg white stimulation, to a similar extent in BER and BET groups, while other interferon receptors were not regulated or different between groups (data not shown). No differences in IFNα or IFNγ secretion were observed between these two groups, although the 5 day post-stimulation timepoint chosen for optimal T cell-derived cytokine production may not be optimal, particularly for the innate cytokine IFNα.

### Immune Annotation of Egg-Induced DEG Signatures

**Fig 5:**
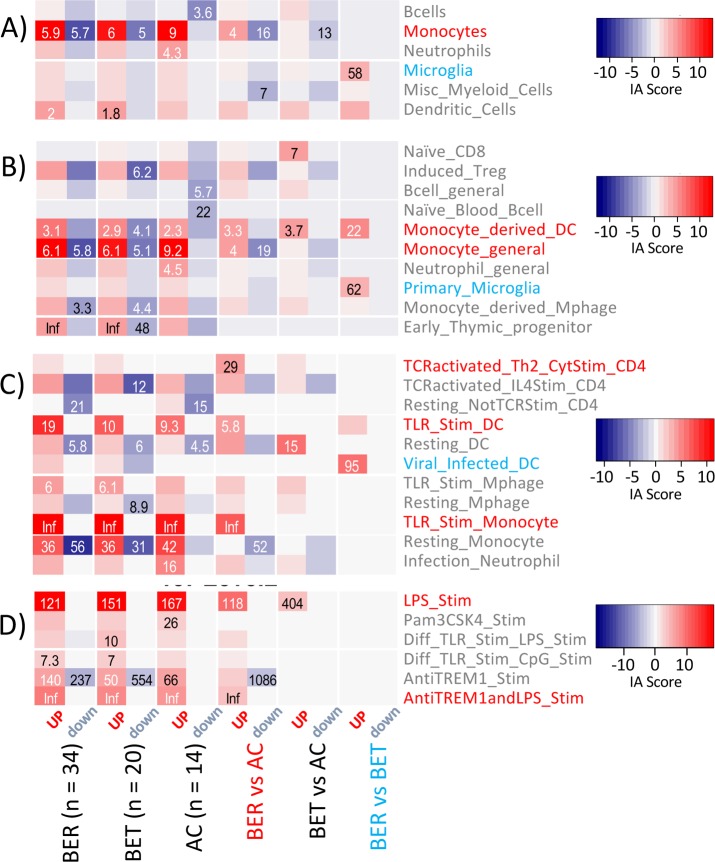
Immune Annotation analysis of the differentially expressed gene signatures. The color intensity indicates the Immune Annotation Score and the values are the odds ratios from Fisher’s exact tests with BH adjusted p-value < 0.1 for the given test. A. Level 1 (Cells), B. Level 2 (Cell Subsets), C. Level 3 (Activation Status), D. Level 4 (TLR-activated myeloid cells). The color indicates whether the genes in the signature were up-regulated (red) or down-regulated (blue). The results are shown for twelve of the total 24 DEG signatures used in the analysis, and immune Annotation categories with at least a single category with BH adjusted p-value ≤ 0.1. Colored text highlights significant associations with the matching DEG signature. Inf = Infinite, all observations were within the subgroup.

Utilization of publicly available resources for functional annotation identifies general biological processes or specific signaling pathways, but does not provide cell type-specific information. Transcriptional signatures for a number of relevant human cell types are available through Differentiation Map Portal (DMAP), but these include only eight different T-cell subsets, four NK cell subsets, six different B-cell populations, and a small number of myeloid cell types without any activation state variation. Immunological Genome Project (ImmGen) provides transcriptional data for a great number of immune cell subsets with different activation states, but only for murine cells. In order to be able to utilize transcriptional signatures representing a large variety of cell types and activation states, we generated a novel functional annotation resource, Immune Annotation, expanding on the MSigDB immunologic signature dataset comprised of 1910 GEO derived signatures derived from human and mouse experimental data. Signatures were annotated into categories by cell type (Level 1, 16 categories), cell subsets (Level 2, 130 categories), cell activation status (Level 3, 160 categories), and myeloid-focused TLR activation (Level 4, 22 categories). Importantly, all differential expression used paired samples in which egg-stimulated cells were compared to unstimulated cells, controlling for any possible differences in cell populations. The most significant results from Immune Annotation application to EW versus M DEG signatures are presented in **Fig 5**. Only categories with significant associations are shown. Among the Level 2 categories, induced Tregs had a high Immune Annotation Score within the down-regulated gene signature comparing BER or BET to AC. At Level 3, focusing on the activation state of immune cell types, multiple categories representing signatures of TLR-stimulated myeloid cells were enriched in the up-regulated BER_(EWvs.M)_vs.AC_(EWvs.M)_ signature. Focusing on activation states of myeloid cells in Level 4, signatures representing TLR4-mediated myeloid cell activation, but not activation via TLR3, TLR1/TLR2, TLR9 or TLR7, were significantly enriched in the BER and BET vs. AC DEG signatures. Therefore, egg stimulation of PBMCs from egg allergic subjects uniquely evokes gene transcription consistent with TLR4 activated myeloid cells. Interestingly, when comparing the BER vs. BET signature, the “Viral Infected DCs” category in Level 3 had a high Immune Annotation Score, consistent with the previous pathway analysis showing a significant enrichment of IFNγ and α response genes in the BER vs. BET signature. Together, these data raise the possibility that immune tolerance to baked egg may be associated with negative regulation of interferon response genes in DCs and perhaps other cell populations.

### Identification of Co-Expressed Gene Modules

**Fig 6:**
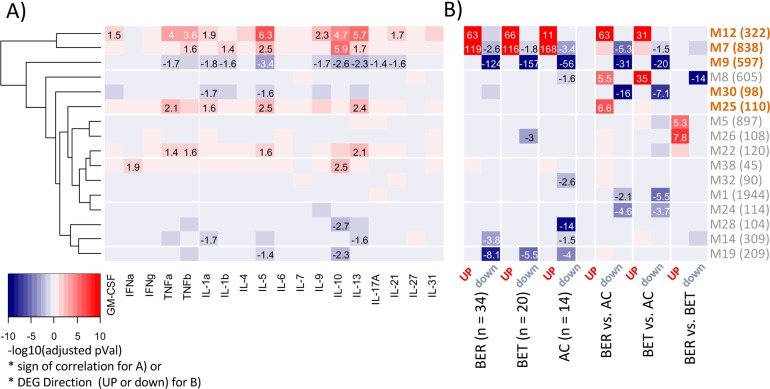
Relationships between co-expression modules, cytokines, and differentially expressed gene signatures. **A**) Association between gene modules and cytokine production, with the color intensity indicating p-value (–log10, BH-adjusted) from linear regression between the modules’ eigengenes and the level of cytokine, and color indicating the correlation direction (red = positive, blue = negative). **B)** Enrichment of the co-expression modules in differentially expressed gene signatures, with the color intensity indicating p-value (–log10, BH-adjusted) from Fisher’s exact test for modules’ enrichments in the DEG signatures, and color indicating whether the genes in the signature were up-regulated (red) or down-regulated (blue). Module size (number of genes) is indicated in parenthesis after the label; the color scale (p-value) is truncated at ±10 (max is 163).

In addition to generating lists of differentially expressed genes, high throughput transcriptome data can be utilized to identify groups of genes with shared expression patterns (modules) by generating a co-expression network in a non-supervised approach. This approach assumes that a shared expression pattern implies shared function, reducing the complexity of the entire transcriptome to a limited number of co-expressing modules that can be annotated to shed light on function. Using a widely utilized method of weighted correlation network analysis (WGCNA)[11] we created a co-expression network from 105 samples from the three clinical groups with and without EW stimulation, for which both transcription and cytokine data were available. A total of 39 co-expression modules (size ranging between 45 and 1944 genes) were identified and named numerically in order of module size. We then tested the association of these gene modules with cytokine secretion and differential gene expression (**Fig 6**).

We identified five modules (M7, M12, M22, M25, and M38) that were significantly correlated with increased expression of at least one of the measured cytokines (**Fig 6A**). M7 and M38 were positively correlated with IL-10, suggesting a potential regulatory role of these gene modules, while M12, M22, and M25 were positively correlated with a Th2 pattern of cytokine production. Another five modules (M9, M14, M19, M28, and M30) were negatively correlated with at least one cytokine. M19 and M28 modules were negatively correlated with IL-10, while M9, M14, and M30 were negatively correlated with Th2 cytokines.

To assess module association with gene signatures, again, we used standardized DEG signatures across conditions. Out of 39 identified modules, 29 had significant enrichment (after FDR correction) with at least one DEG signature (**Fig 6B**). We identified a total of five modules (M7, M9, M12, M25, and M30) that were associated with both DEG signatures and cytokine production, and we focused on these modules further.

### Functional Annotation of Gene Modules

**Fig 7:**
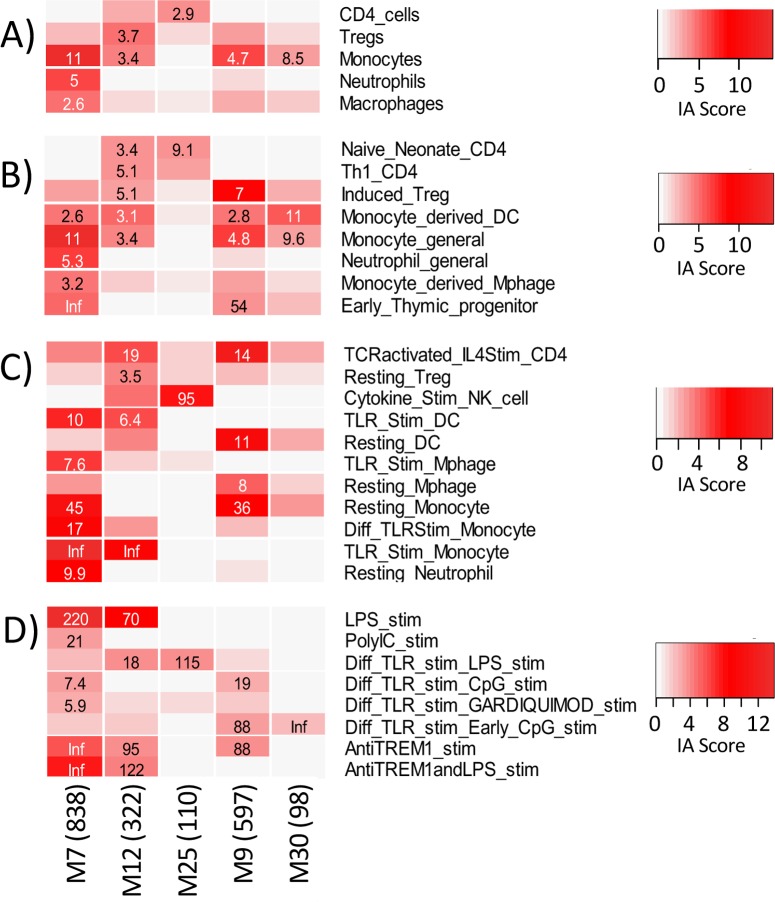
Immune Annotation enrichment in five egg allergy-associated co-expression modules. Enrichment results are shown for A) Level 1 immune cell types; B) Level 2 immune cell sub-types; C) Level 3 activation state of immune cell types; and D) Level 4 myeloid cell stimulation. The color intensity indicates the Immune Annotation Score and the values are the Fisher’s exact tests’ Odds Ratios for tests with BH adjusted p-value ≤ 0.10. Only categories with at least one significant result are included. Inf = Infinite, all observations were within the subgroup.

Immune Annotation analysis was performed with focus on the five gene modules (M12, M25, M7, M9, and M30) associated with both differential gene expression and cytokine production in allergic subjects (**Fig 7**). Two of the modules (M12, M9) were significantly associated with signatures of IL-4-stimulated Th2 cells, an expected finding that provides support that this bioinformatics approach yields biologically relevant associations. In addition to this association, several novel associations were found. The M12 module was associated with TLR-stimulated monocytes and DCs, and with LPS and anti-TREM1 stimulation of myeloid cells. M25 was strongly associated with cytokine-stimulated NK cells, and also LPS-specific stimulation of myeloid cells. The M7 module was significantly enriched for multiple categories representing signatures of myeloid cells, in particular TLR activated DCs. M9, which negatively correlated with Th2 cytokine production and egg-induced gene expression in allergic subjects, was significantly enriched for categories representing induced Tregs. The M30 module was enriched for categories representing signatures of monocytes and CpG-stimulated myeloid cells. Only categories with significant associations are shown in Fig 7.

In summary, functional enrichment analysis of either differential gene expression signatures or co-expressed gene modules identified signatures of four main cell types positively or negatively associated with egg-induced gene transcription: Th2 cells, induced Tregs, NK cells, and a strong and consistent association with TLR4-activated myeloid cells.

## Discussion

Our data demonstrate that transcriptional profiling of bulk PBMCs is a useful tool that, when combined with an integrative informatics analysis approach, can identify novel biological processes relevant to egg allergy. Using a combination of differential gene expression and multiplex cytokine measurement, we observed that egg allergy was associated with IL-9, IL-5, and TNFα expression and secretion, as well as expression of CEACAM1, and CISH. These data are consistent with a previous report by Brough et al [12], demonstrating increased IL-9, IL-5, CEAMCAM1, CISH expression in purified peanut-responsive CD4+ T cells from peanut allergic subjects compared to atopic controls. Based on frequency of allergen-specific T cells in peripheral blood reported in other studies [15] and our own unpublished work, we would expect approximately 100–300 egg-responsive cells for every million CD4+ T cells. Therefore, changes in those few egg-responsive CD4+ T cells would need to be substantial to be observed when diluted out with transcripts from antigen non-responsive cells. We were therefore pleased to observe differentially expressed genes in response to egg white, including cytokines consistent with pro-inflammatory Th2 or Th9 cells. Our simple approach, requiring relatively little blood sample volume and laboratory sophistication, therefore can generate data that reflects the allergen-specific CD4+ T cell response. In addition, the similarity in gene expression profiles between egg allergic subjects reported here and that previously published from peanut allergic subjects suggests a similar mechanism underlying these food allergies. Future studies of CD4+ T cells in egg-allergic individuals will determine whether the IL-9 is being produced by Th9 or Th2 cells, and highlights the potentially central role of IL-9 in food allergy as indicated by studies in mouse models [16–18].

An important advantage of a bulk PBMC-derived expression dataset is that it contains expression data from multiple cell types. Utilization of the co-expression network and functional annotation approaches allowed us to identify co-expressed groups of genes (modules) with shared functions both across multiple cell types and specific to a particular cell type. In conjunction with the Immune Annotation resource, developed for the current project, this approach allowed us to associate differentially expressed genes, or co-expressed gene modules, with specific immune cell types or particular activation states, and extended our capacity to link activity of individual cell types with egg allergy. Functional enrichment using either co-expressed gene modules or differentially expressed genes identified signatures representing Th2 cells, induced Tregs, myeloid cells, and NK cells. Th2 cells and Tregs are already the focus of investigation in studies of food allergy, including the study from which these samples were obtained (CoFAR7). Mouse models have identified a critical role for Tregs in the suppression of food allergy and a role for “reprogrammed” Th2 cytokine-producing Tregs in driving food allergy [19, 20]. There is evidence that Tregs are expanded during natural resolution of food allergy [21], [7], [22]. By enrichment analysis, we observed a negative association of signatures representing Treg-associated gene transcription with egg allergy, which along with the positive association with signatures representing Th2 cells, provides support that our computational approach to the study of food allergy can identify biologically meaningful processes.

In addition to Tregs and Th2 cells, our analysis pointed to a novel contribution of myeloid cells (monocytes, macrophages, and dendritic cells) and NK cells to egg-induced gene expression in allergic individuals. Human DCs express a form of the high-affinity IgE receptor FcεRI and therefore have the potential to respond directly to allergen through allergen-specific IgE [23]. Myeloid cells also express a number of Fcγ receptors, showing potential for activation by IgG/allergen immune complexes. There is evidence that DCs from food allergic subjects respond differently to allergen stimulation than DCs from non-affected siblings [24]. Enrichment analysis strongly identified signatures of TLR stimulation of myeloid cells, and specifically TLR4, as overlapping with egg-induced differential gene expression in allergic individuals. A role for TLR4 in the allergic response to egg was unexpected, and unlikely to be merely due to LPS contamination since egg extracts used for stimulation were cleaned of endotoxin prior to use. Furthermore, the enrichment of signatures representing TLR4-mediated stimulation was unique to the response to egg in egg allergic individuals. Previous studies have implicated the TLR4 signaling pathway in allergic responses. TLR4 contributes to innate immune responses driving sensitization to dust mite and nickel, and TLR4 signaling is critical to in vivo responsiveness to these allergens [25, 26]. Der p 2 and Der f 2 (from dust mite), Fel d 1 (cat allergen) and Can f 6 (dog allergen) are lipid-binding proteins that enhance signaling through TLR4 [27]. A recent report studying the immune profile of cord blood samples demonstrated that monocyte responsiveness to LPS was significantly increased in infants who developed food allergy, and that monocyte-derived inflammatory cytokines could promote the development of Th2 cells [28]. Therefore, there may be a unique contribution of TLR, and TLR4 associated pathways particularly, in the generation of inappropriate immune responses to egg in allergic individuals.

The primary objective of this study was to examine transcriptional differences in the response to egg between egg allergic subjects who did or did not tolerate extensively heated egg. Although the transcriptional differences observed between these two groups were relatively small, pathway analysis of differentially expressed genes identified genes downstream of both Type I interferons and IFNγ as being uniquely downregulated in response to egg stimulation in BET subjects. Immune annotation analysis highlighted genes associated with virally infected DCs, suggesting that there may be unique phenotypic changes in the DC populations in baked egg tolerant subjects. Cross-talk between anti-viral responses or TLR signaling and IgE signaling in DCs has been shown [29, 30], and may be a potential pathway regulating the development of immune tolerance. Further mechanistic studies are needed to dissect the role of this pathway, including cell types involved, in tolerance to baked egg. Factors that could modulate this pathway and promote tolerance could potentially be utilized for therapeutic purpose.

Our results may be used in at least three future approaches. The identification of differentially expressed single genes (i.e. IL-9, TNFα) could be used for selective targeting during therapy. The identification of novel processes, such as TLR4-activated myeloid cells in egg allergy or IFN related pathways in baked egg tolerance, prompts further study using traditional experimental approaches that may change our understanding of the immunology of food allergy. And finally, a transcriptional signature of the aberrant response to egg, and common with other food allergies including peanut, could be used to identify drugs with opposing actions, a computational approach to drug repurposing that has led to discovery of new therapeutics for diseases such as inflammatory bowel disease [31–33].
